# Supplementary material for: Integrated analysis of the aqueous humor microbiome and lens capsule transcriptome in high myopia cataract: a pilot study
Source: Front Med (Lausanne). 2026 Jun 16;13:1845205. doi: 10.3389/fmed.2026.1845205 (PMC13314463; doi:10.3389/fmed.2026.1845205)
Supplement: Supplementary file 3 [file Data_Sheet_1.zip › 3.Beta_Diversity/PCoA/C372089/PCoA_euclidean_3D.pdf]

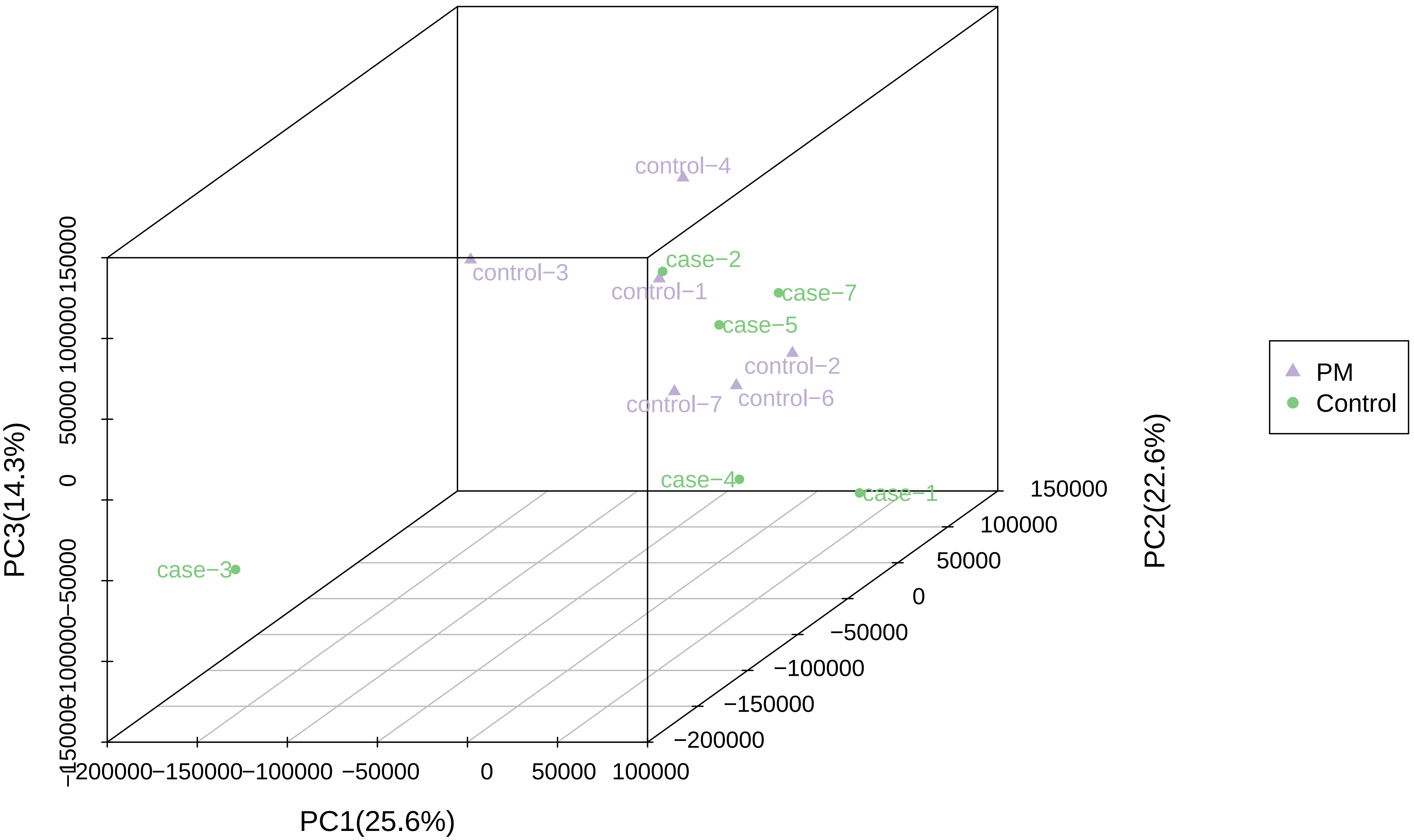
control−4
control−3
case−2
control−1
case−7
case−5
control−2
control−6
control−7
case−4
case−1
case−3
PM
Control
PC1(25.6%)
PC2(22.6%)
PC3(14.3%)
−200000 −150000 −100000 −50000 0 50000 100000
−200000 −150000 −100000 −50000 0 50000 100000 150000
−150000 −100000 −50000 0 50000 100000 150000
